# Intergovernmental engagement on health impacts of climate change

**DOI:** 10.2471/BLT.20.270033

**Published:** 2020-11-04

**Authors:** Niheer Dasandi, Hilary Graham, Pete Lampard, Slava Jankin Mikhaylov

**Affiliations:** a International Development Department, University of Birmingham, 1143 Muirhead Tower, Birmingham B15 2TT, England.; b Department of Health Sciences, University of York, York, England.; c Data Science Lab, Hertie School, Berlin, Germany.

## Abstract

**Objective:**

To examine countries’ engagement with the health impacts of climate change in their formal statements to intergovernmental organizations, and the factors driving engagement.

**Methods:**

We obtained the texts of countries’ annual statements in United Nations (UN) general debates from 2000 to 2019 and their nationally determined contributions at the Paris Agreement in 2016. To measure countries’ engagement, we used a keyword-in-context text search with relevant search terms to count the total number of references to the relationship of health to climate change. We used a machine learning model (random forest predictions) to identify the most important country-level predictors of engagement. The predictors included political and economic factors, health outcomes, climate change-related variables and membership of political negotiating groups in the UN.

**Findings:**

For both UN general debate statements and nationally determined contributions, low- and middle-income countries discussed the health impacts of climate change much more than did high-income countries. The most important predictors of engagement were health outcomes (infant mortality, maternal deaths, life expectancy), countries’ income levels (gross domestic product per capita), and fossil fuel consumption. Membership of political negotiating groups (such as the Group of 77 and Small Island Developing States) was a less important predictor.

**Conclusion:**

Our analysis indicated a higher engagement in countries that carry the heaviest climate-related health burdens, but lack necessary resources to address the impacts of climate change. These countries are shouldering responsibility for reminding the global community of the implications of climate change for people’s health.

## Résumé

**Objectif:**

Étudier l'engagement des pays vis-à-vis de l'impact sanitaire du changement climatique au travers de leurs déclarations officielles aux organisations intergouvernementales, et les facteurs qui sous-tendent cet engagement.

**Méthodes:**

Nous nous sommes procuré les textes des déclarations annuelles des pays au débat général de l'Organisation des Nations Unies (ONU) entre 2000 et 2019, ainsi que les contributions à l'Accord de Paris déterminées au niveau national en 2016. Pour évaluer l'engagement de chacun d'eux, nous avons mené une recherche par mots-clés en contexte avec des termes pertinents qui nous ont permis de compter le nombre total de références aux liens établis entre santé et changement climatique. Nous avons employé un modèle d'apprentissage automatique (prédictions par forêts aléatoires) afin d'identifier les principaux indicateurs d'engagement à l'échelle nationale. Parmi ces indicateurs figuraient notamment les facteurs économiques et politiques, les conséquences pour la santé, les variables relatives au changement climatique et l'appartenance aux groupes de négociation politique au sein de l'ONU.

**Résultats:**

Tant pour les déclarations lors des débats de l'ONU que pour les contributions fixées au niveau national, les pays à faible et moyen revenu ont nettement plus souvent abordé l'impact sanitaire du changement climatique que les pays à haut revenu. Les indicateurs d'engagement majeurs étaient les conséquences pour la santé (mortalité infantile, décès maternels, espérance de vie), le niveau de revenu des pays (produit intérieur brut par habitant) et la consommation d'énergie fossile. En revanche, la participation aux groupes de négociation politique (tels que le Groupe des 77 et les petits États insulaires en développement) a eu moins de poids.

**Conclusion:**

Notre analyse révèle un plus grand engagement dans les pays qui paient le plus lourd tribut au changement climatique, mais ne possèdent pas les ressources nécessaires pour faire face à l'impact qu'il exerce. Ces pays assument la responsabilité de rappeler à la communauté internationale quelles sont les répercussions du changement climatique sur la santé des populations.

## Resumen

**Objetivo:**

Analizar la participación de los países en los efectos del cambio climático sobre la salud en sus declaraciones oficiales ante las organizaciones intergubernamentales, así como los factores que motivan esa participación.

**Métodos:**

Se obtuvieron los textos de las declaraciones anuales de los países en los debates generales de las Naciones Unidas (ONU) de 2000 a 2019 y sus contribuciones establecidas a nivel nacional en el Acuerdo de París en 2016. Para medir la participación de los países, se empleó una búsqueda de texto de palabras clave en el contexto con los términos de búsqueda pertinentes para contar la cantidad total de referencias a los vínculos entre la salud y el cambio climático. Se usó un modelo de aprendizaje automático (predicciones de los bosques aleatorios) para identificar los indicadores más importantes de la participación de los países. Los indicadores incluían factores políticos y económicos, resultados sanitarios, variables del cambio climático y la pertenencia a los grupos de negociación política de las Naciones Unidas.

**Resultados:**

Tanto en las declaraciones del debate general de las Naciones Unidas como en las contribuciones establecidas a nivel nacional, los países de ingresos medios y bajos debatieron los efectos del cambio climático en la salud mucho más que los países de ingresos altos. Los indicadores más importantes de la participación fueron los resultados sanitarios (mortalidad infantil, mortalidad materna, esperanza de vida), los niveles de ingresos de los países (producto interno bruto per cápita) y el consumo de combustibles fósiles. La pertenencia a los grupos de negociación política (como el Grupo de los 77 y los Pequeños Estados Insulares en Desarrollo) fue el indicador de menor importancia.

**Conclusión:**

El análisis de este estudio indica que hay una mayor participación de los países que soportan las mayores cargas sanitarias relacionadas con el clima, pero que carecen de los recursos necesarios para resolver los efectos del cambio climático. Estos países asumen la responsabilidad de recordar a la comunidad mundial sobre las consecuencias del cambio climático para la salud de las personas.

## ملخص

الغرض فحص مشاركة الدول في الآثار الصحية لتغير المناخ في بياناتها الرسمية إلى المنظمات الحكومية الدولية، والعوامل التي تدفع إلى المشاركة.

الطريقة حصلنا على نصوص البيانات السنوية للدول في المناقشات العامة للأمم المتحدة (UN) من عام 2000 إلى عام 2019، ومساهماتها المحددة على المستوى الوطني في اتفاقية باريس في عام 2016. لقياس مشاركة الدول، استخدمنا البحث عن نص باستخدام الكلمات المفتاحية المتعلقة بالسياق مع مصطلحات البحث ذات الصلة لحساب العدد الإجمالي للإشارات إلى علاقة الصحة بالتغير في المناخ. استخدمنا نموذج التعلم الآلي (التنبؤات المتشعبة العشوائية) لتحديد أهم مؤشرات التنبؤ بالمشاركة على مستوى الدولة. شملت مؤشرات التنبؤ العوامل السياسية والاقتصادية، والنتائج الصحية، والمتغيرات المتعلقة بتغير المناخ، وعضوية مجموعات التفاوض السياسي في الأمم المتحدة.

النتائج بالنسبة لكل من بيانات المناقشة العامة للأمم المتحدة، والمساهمات المحددة على المستوى الوطني، ناقشت الدول ذات الدخل المنخفض والدخل المتوسط الدخل الآثار الصحية لتغير المناخ بشكل أكثر بكثير مما قامت به الدول ذات الدخل المرتفع. كانت أهم مؤشرات التنبؤ بالمشاركة هي النتائج الصحية (وفيات المواليد، ووفيات الأمهات، ومتوسط العمر المتوقع)، ومستويات دخل الدول (الناتج المحلي الإجمالي للفرد)، واستهلاك الوقود الأحفوري. كانت عضوية مجموعات التفاوض السياسية (مثل مجموعة الـ 77 والدول الجزرية الصغيرة النامية) مؤشرًا أقل أهمية للتنبؤ.

الاستنتاج أشار تحليلنا إلى مشاركة أكبر في الدول التي تتحمل أثقل الأعباء الصحية المتعلقة بالمناخ، ولكنها تفتقر إلى الموارد الضرورية لمعالجة آثار تغير المناخ. يقع على عاتق هذه الدول مسؤولية تذكير المجتمع العالمي بآثار تغير المناخ على صحة الناس.

## 摘要

**目的:**

考察各国在向政府间组织提交的正式声明中就气候变化对健康的影响这个问题报告的参与情况,以及推动参与的因素。

**方法:**

我们获得了 2000 至 2019 年联合国 (UN) 一般性辩论中各国的年度声明文本,以及 2016 年《巴黎协定》中各国自主贡献的文本。为了衡量各国的参与情况,我们使用相关搜索词进行上下文关键字文本搜索,以计算有关健康与气候变化之间关系的参考文献的总数。我们使用机器学习模式(随机森林预测)来确定国家层面有关参与情况的最重要预测因素。预测因素包括了政治和经济因素、健康结果、气候改变相关变量和联合国政治谈判小组的成员资格。

**结果:**

在联合国一般性辩论声明和国家自主贡献的调查结果中,中低收入国家针对气候变化对健康的影响的讨论远远超过高收入国家。参与情况的最重要预测因素是健康结果(婴儿死亡率、孕产妇死亡率、预期寿命)、国家收入水平(人均国内生产总值)和化石燃料消耗。政治谈判小组的成员资格(例如七十七国集团和小岛屿发展中国家)是次要预测因素。

**结论:**

我们的分析表明,承担气候相关健康负担最重的国家参与度更高,但是它们缺乏必要的资源来解决气候变化的影响。这些国家肩负着提醒国际社会气候变化对人们健康产生的影响的责任。

## Резюме

**Цель:**

Изучить вовлеченность стран в решение проблем, связанных с воздействием изменения климата на здоровье человека, на основании официальных заявлений в адрес межправительственных организаций, а также факторы, способствующие такому участию.

**Методы:**

Авторы получили тексты ежегодных заявлений стран в ходе общеполитических дискуссий Организации Объединенных Наций (ООН) с 2000 по 2019 год, а также данные об определяемых на национальном уровне вкладах по Парижскому соглашению 2016 года. Для измерения степени вовлеченности стран авторы использовали текстовый поиск по ключевым словам в контексте в сочетании с соответствующими критериями поиска, чтобы подсчитать общее количество ссылок на взаимосвязь между здоровьем человека и изменением климата. Авторы использовали модель машинного обучения (прогнозирование на основе случайного леса), чтобы определить наиболее важные предикторы вовлеченности стран. Предикторы включали в себя политические и экономические факторы, показатели здоровья, переменные, связанные с изменением климата, а также членство в политических переговорных группах ООН.

**Результаты:**

Как в заявлениях стран в рамках общеполитических дискуссий ООН, так и в определяемых на национальном уровне вкладах страны с низким и средним уровнем дохода обсуждали последствия изменения климата для здоровья человека намного чаще, чем страны с высоким уровнем дохода. Наиболее важными предикторами вовлеченности были показатели здоровья (младенческая смертность, материнская смертность, ожидаемая продолжительность жизни), уровень доходов страны (валовой внутренний продукт на душу населения) и потребление ископаемых видов топлива. Членство в политических переговорных группах (таких как Группа 77 и Альянс малых островных развивающихся государств) являлось менее важным предиктором.

**Вывод:**

Проведенный анализ показал более высокую вовлеченность стран, несущих самое тяжелое бремя в области здравоохранения, связанное с изменением климата, но не располагающих необходимыми ресурсами для борьбы с последствиями изменения климата. Эти страны приняли на себя огромную ответственность за то, чтобы напомнить мировому сообществу о последствиях изменения климата для здоровья человека.

## Introduction

Climate change is taking an increasing toll on people’s health. The increase in heatwaves, drought, floods and other climate hazards is increasing the risk of climate-related illness and death as well as reversing gains made in reducing food insecurity and global hunger.^1^^,^^2^ Air pollution, primarily driven by fossil fuel emissions, is the major environmental risk factor for premature death and has impacts on child health and survival.^3^^–^^5^ Highlighting these human impacts is seen as a way of accelerating climate action, by mobilizing public and political support within countries and by building alliances between countries.^6^^,^^7^ While not without its challenges, a health framing of climate change may have the potential to overcome geopolitical divisions that have hindered international efforts towards mitigating climate change.^8^^,^^9^

A key arena for global engagement is the United Nations (UN), the largest and most influential intergovernmental organization in the world.^10^ Although the UN includes a range of structures that enable governments to engage with health and climate change, we know little about the extent to which countries do so. Studies that have considered this issue tend to find major differences in countries’ levels of engagement. For example, previous studies point to generally low levels of government engagement with climate change and health in the UN General Assembly, with the Small Island Developing States driving engagement.^1^ Studies of the nationally determined contributions, which countries submit as part of the Paris Agreement (see below), suggest that, while most countries reference health, few nationally determined contributions included detailed discussions of health plans.^11^^–^^13^ This finding has led some commentators to argue that health remains peripheral to climate change politics.^14^

In this study, we examined countries’ engagement with the health impacts of climate change in their formal statements to intergovernmental organizations, and the factors that drive engagement. Building on earlier studies,^12^^,^^13^^,^^15^ we based our study on texts from the general debate of the UN General Assembly and nationally determined contributions pledges, which are part of the Paris Agreement negotiated at the 21st Conference of the Parties of the UN Framework Convention on Climate Change.^16^^,^^17^ The UN general debate takes place annually, and offers a platform for all UN Member States to address the UN General Assembly and discuss various issues in world politics, ranging from international security to climate change.^18^ The sessions are widely seen as an indicator of international opinion on important issues, even those not on the agenda for a particular session.^19^ In contrast, the nationally determined contributions are directly related to international climate change negotiations. As part of the Paris Agreement, countries decide on their own contributions towards achieving a low-emissions pathway and climate-resilient development, set out in their nationally determined contributions, to be reviewed and revised on a 5-yearly cycle.^20^ The flexible structure of the nationally determined contributions gives countries the opportunity to include other policy priorities relevant to emissions reduction and adaptation planning, including public health priorities.^21^ This flexible structure means that the pledges are influenced by wider political processes, and hence can be understood as political texts that shed light on differences and tensions between countries with respect to climate policy.^22^

Governments can discuss health and climate change in other international fora, such as the World Health Assembly and the UN Framework Convention on Climate Change Conference of the Parties. However, not all countries deliver statements in these fora, whereas the UN general debate and nationally determined contributions cover all UN Member States. In this study we aimed to describe the extent to which a health framing of climate change avoids the geopolitical divisions that have hindered international efforts to address climate change.^23^ For example, we considered whether political groups in the UN influence engagement with the health impacts of climate change. The influence of such groups in the UN General Assembly has long been recognized.^24^ Similarly, countries exert leverage in UN Framework Convention on Climate Change negotiations through membership of such groups as the Group of 77 (G77) at the UN, the European Union (EU), the Least Developed Countries and Small Island Developing States.^25^ Our analytical framework also took account of a broad range of country-level factors which may influence engagement with the health impacts of climate change: economic, social and political characteristics and health profile. 

## Methods

### Data sources

Our data for the analysis of UN general debate speeches came from the UN General Debate Corpus, a data set which contains the transcripts of annual statements of all Member States, in a document format that is pre-processed and prepared for the application of natural language processing to the official English versions of statements.^20^ We examined statements for 2000–2019, as before 2000 there were few references to the health impacts of climate change. In total, we analysed 3829 statements from the 20-year period, consisting of 310 425 sentences and 8 350 632 words (available in the data repository).^26^

We also collected the texts of countries’ nationally determined contributions from the UN Framework Convention on Climate Change registry.^27^ As of January 2020, 185 countries had submitted their first nationally determined contributions (EU Member States produce a joint nationally determined contribution), with the majority of these produced in 2016. We examined 158 documents, consisting of 25 237 sentences and 862 724 words (data repository).^26^

### Search strategy

We applied an automated keyword-in-context search to the two sets of texts, which enabled us to find the frequency of a term in texts, with the words appearing immediately before and after the search term.^28^ For the nationally determined contributions, this search was based on producing a set of health-related terms (Box 1). Our measure of engagement was based on the frequency of these terms appearing in the texts of countries’ pledges. We employed a similar approach in measuring engagement in the transcripts of statements in the UN general debate. However, as references to health in the statements may be unrelated to climate change, we used health-related terms and a set of climate change-related terms (Box 1). Both the health- and climate change-related terms were derived from The Lancet Countdown on health and climate change analysis of government engagement with climate change and health.^1^ To measure engagement in general debate statements, we searched the 25 words immediately before and after a health-related term to see whether any of the climate change-related terms appeared close to a mention of health. We discuss these approaches further in the data repository.^26^

Box 1 **Key search terms on health and climate change used in the study of country engagement with the health impacts of climate change**

**Health terms:**

malaria, diarrhoea, infection, disease, diseases, SARS, measles, pneumonia, epidemic, epidemics, pandemic, pandemics, epidemiology, health care, health, mortality, morbidity, nutrition, illness, illnesses, NCD, NCDs, air pollution, nutrition, malnutrition, malnourishment, mental disorder, mental disorders, stunting.

**Climate change terms:**

climate change, changing climate, climate emergency, climate action, climate crisis, climate decay, global warming, green house, temperature, extreme weather, global environmental change, climate variability, greenhouse, greenhouse-gas, low carbon, GHGE, GHGEs, renewable energy, carbon emission, carbon emissions, carbon dioxide, carbon-dioxide, CO_2_ emission, CO_2_ emissions, climate pollutant, climate pollutants, decarbonization, decarbonisation, carbon neutral, carbon-neutral, carbon neutrality, climate neutrality, net-zero, net zero.

### Selection of predictors

To analyse factors associated with countries’ higher and lower levels of engagement with climate change and health, we included a range of predictors in our models (Table 1). The predictors included a country’s economic measures (gross domestic product (GDP) per capita, economic growth, level of trade, access to electricity); political indicators (level of democracy); and geographical and demographic factors (population size, latitude). Our analysis also included several population health-related predictors (life expectancy, infant mortality rate and risk of maternal death; a predictor for the population’s healthy life years; and country’s expenditure on health). We included several predictors associated with climate change (carbon dioxide (CO_2_) emissions, fossil fuel energy consumption, renewable energy consumption; ecological footprint of consumption measured in global hectares or carbon footprint; and oil rents). Finally, we included predictors based on countries’ participation in important negotiating groups in the UN General Assembly and the UN Framework Convention on Climate Change.^28^ The groups included the G77, the African Group of Negotiators, the Arab States, the EU, the Least Developed Countries, the Small Island Developing States, the Umbrella Group and the Environmental Integrity Group.

**Table 1:** Description of predictors and data sources used in the study of countries’ engagement with the health impacts of climate change

Predictor	Description	Data source
**Economic factors**		
GDP per capita	Logarithm of GDP per capita in constant 2010 United States dollars	World Bank World Development Indicators^29^
Economic growth	Annual percentage growth rate of GDP based on local market prices	World Bank World Development Indicators^29^
Trade (% of GDP)	Total sum of exports and imports as a proportion of GDP	World Bank World Development Indicators^29^
Access to electricity (% of population)	Percentage of population that has access to electricity	World Bank World Development Indicators^29^
**Political factors**		
Democracy	Combination of Freedom House and Polity democracy scores	Hadenius & Teorell, 2005^30^
**Geographical and demographic factors**		
Population size	Logarithm of the total population of the country	World Bank World Development Indicators^29^
Latitude	Absolute value of the distance of the capital city from the equator	World Bank World Development Indicators^29^
**Health-related factors**		
Life expectancy	Number of years a newborn infant would live if the prevailing patterns of mortality at the time of birth were to remain the same throughout its life	World Bank World Development Indicators^29^
Infant mortality rate	Number of infants dying before reaching 1 year of age per 1000 live births	World Bank World Development Indicators^29^
Risk of maternal death	Probability that a 15-year-old female will die eventually from a maternal cause, assuming that current levels of fertility and mortality do not change	World Bank World Development Indicators^29^
Healthy life years	Years lived in less than ideal health and years lost due to premature mortality	Institute for Health Metrics and Evaluation Global Burden of Disease study^31^
Health expenditure (% of GDP)	Current level of health expenditure as a proportion of GDP	World Bank World Development Indicators^29^
**Climate change-related factors **		
CO_2_ emissions	CO_2_ emissions from burning fossil fuels and manufacturing cement in metric tonnes per capita	World Bank World Development Indicators^29^
Fossil fuel energy consumption (% of total energy consumption)	Share of coal, oil, petroleum and natural gas products in total energy consumption	World Bank World Development Indicators^29^
Renewable energy consumption (% of total energy consumption)	Share of renewable energy in total energy consumption	World Bank World Development Indicators^29^
Carbon footprint	Represents the area of forest land required to isolate carbon emissions measured in global hectares per person	Global Footprint Network^32^
Oil rents (% of GDP)	Difference between the value of crude oil production at world prices and total costs of production as a percentage of GDP	World Bank World Development Indicators^29^
**Membership of negotiating groups**		
Group of 77	A group of low-and middle-income countries that was formed to work to establish common negotiating positions in the UN (currently 135 members)	UN Framework Convention on Climate Change^16^^,^^17^
African Group of Negotiators	An alliance of African countries formed to represent the interests of the region in international climate change negotiations. The group was established at the first Convention on Climate Change in 1995 (currently 54 members)	UN Framework Convention on Climate Change^16^^,^^17^
The Arab States	The Member States of the League of Arab States representing the interests of these countries in international climate change negotiations (22 members)	UN Framework Convention on Climate Change^16^^,^^17^
The European Union	The Member States of the European Union (28 countries; 27 countries after 2020)	UN Framework Convention on Climate Change^16^^,^^17^
Least Developed Countries	A group of countries with the lowest indicators of socioeconomic development in the UN system. The group was formed to represent the specific needs and interests of these countries in the UN (currently 48 countries)	UN Framework Convention on Climate Change^16^^,^^17^
Small Island Developing States	A coalition of low-lying small island states that are especially vulnerable to the effects of climate change. The group was formed to emphasize the existential threat that these countries face from climate change and to adopt a common stance in international climate change negotiations (currently 41 countries)	UN Framework Convention on Climate Change^16^^,^^17^
The Umbrella Group	A coalition of countries formed following the adoption of the Kyoto Protocol to represent the interests of non-European Union developed countries in international climate change negotiations (currently 12 countries: Australia, Belarus, Canada, Iceland, Israel, Japan, Kazakhstan, New Zealand, Norway, Russian Federation, Ukraine and United States of America)	UN Framework Convention on Climate Change^16^^,^^17^
The Environmental Integrity Group	A group of countries formed in 2000 to enable countries that did not belong to any bloc (and so were prevented from taking part in negotiations occurring between established groups) to take a constructive role in international climate change negotiations (currently 6 countries: Georgia, Liechtenstein, Mexico, Monaco, Republic of Korea and Switzerland)	UN Framework Convention on Climate Change^16^^,^^17^

CO_2_: carbon dioxide; GDP: gross domestic product; UN: United Nations.

We extracted the data for most of these variables from the World Bank’s World Development Indicators.^29^ Our measure of democracy combined Freedom House and Polity scores.^30^ The data for the healthy life years measure came from the Institute for Health Metrics and Evaluation,^31^ and the measure of countries’ carbon footprint from the Global Footprint Network.^32^ We provide summary statistics for the predictors in the data repository.^26^

### Random forest analysis

We used a machine learning model to identify the most important predictors of country engagement with the health consequences of climate change. Machine learning models differ from standard regression in that they identify the factors that best predict a given outcome, rather than seeking to explain individual relationships between variables. A key advantage of this approach is that it is non-parametric. In addition, high correlation between explanatory variables in standard regression models leads to multicollinearity, which can be avoided in specific machine learning models. Hence, we were able to test a range of predictors even if they were highly correlated, to identify the factors that best predict engagement with the health outcomes of climate change.

We used one of the most widely used machine learning predictive tools – random forests – which are formed of ensembles of decision trees.^33^ The decision tree sorts observations into subgroups by identifying the factor that most accurately separates no engagement with health and climate change from engagement. The tool then identifies additional factors to further reduce the prediction error within each subgroup. Random forests build a large collection of de-correlated trees to improve the quality of prediction. We describe the method in more detail in the data repository.^26^ We implemented the random forest analysis using the *ranger* package of R statistical programming language (R foundation, Vienna, Austria).^34^ We selected the optimal model based on a hyperparameter grid search and did not implement any additional weighting of the features.

## Results

### UN general debate statements

**Fig. 1:**
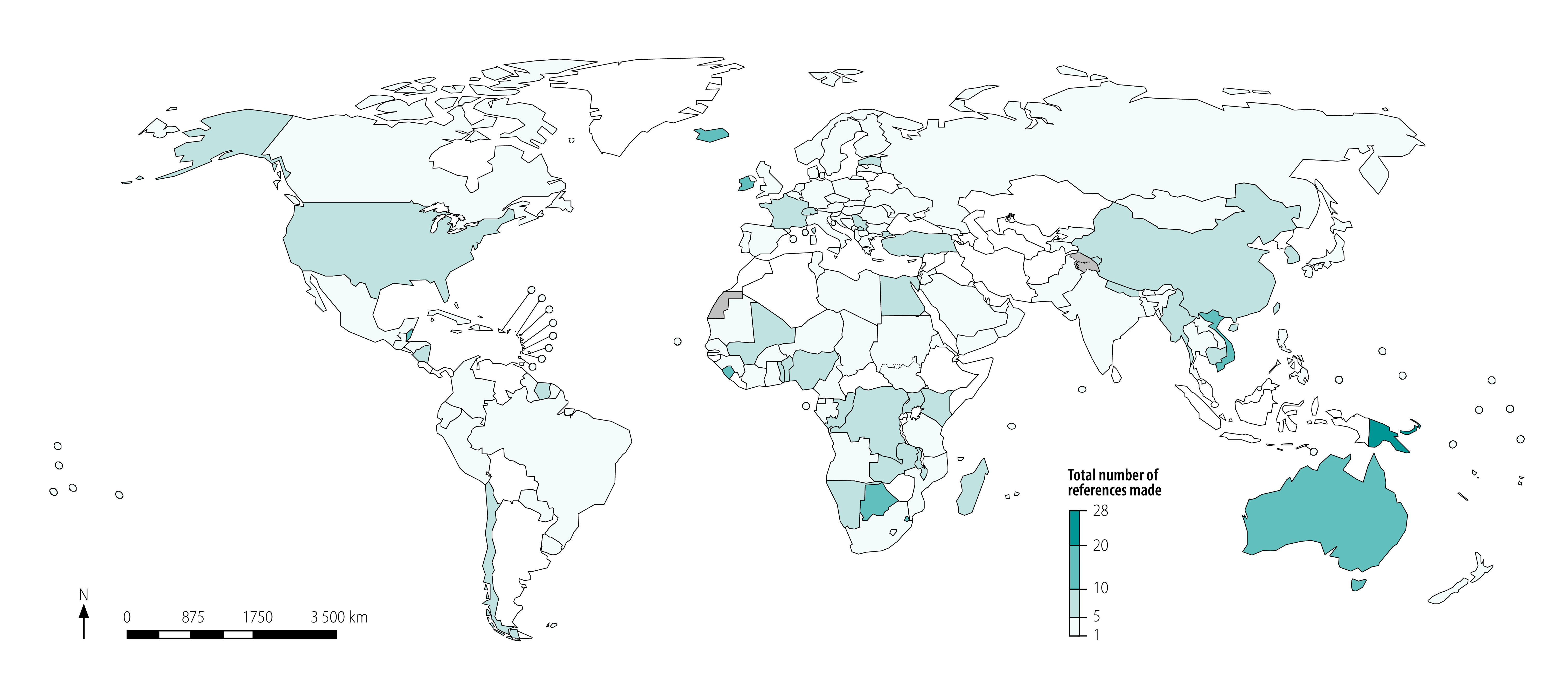
Countries’ references to the health impacts of climate change in speeches at the general debate of the United Nations General Assembly, 2000–2019

We analysed a total of 3860 statements made by 196 countries in the UN general debate over the period 2000–2019. Fig. 1 (available at: http://www.who.int/bulletin/volumes/99/2/20-270033) shows countries grouped according to the total number of references they made about the health impacts of climate change. The references typically highlighted the link between climate change and health or focused on the impacts of climate change on health in a country. For example, Saint Kitts and Nevis’s 2018 UN general debate address described noncommunicable diseases and climate change as “two sides of the same coin,” and Tonga’s 2019 address explains that extreme weather events resulting from climate change are “increasingly more intense… putting the health of our people at risk.”

The results show that overall there has been only modest engagement with the issue of health effects of climate change. However, there were important regional differences. For example, in sub-Saharan Africa, several countries (including Botswana, Eswatini and Sierra Leone) have repeatedly referenced the health impacts of climate change. Several countries in South-East Asia and East Asia (such as China, Myanmar and Viet Nam) also frequently referred to the climate-change–health relationship. The highest engagement was in the Western Pacific Region, notably the Pacific island countries (which cannot be seen on the map due to their small size).

The mean number of references to climate change and health for the full 20-year period was 4.52, with the number of references by countries ranging from 0 to 28. Box 2 lists the 20 countries with the highest number of references to climate change and health and shows that the top five countries were Pacific island countries (ranging from 28 to 22 references). Australia was one of the few high-income countries to frequently refer to the health impacts of climate change (16 references). Australia typically does this in the context of the challenges that the Pacific islands face and the assistance that it provides to help meet the challenges. We found much lower engagement among countries in Europe, such as Germany (4 references), Russian Federation (3 references) and the United Kingdom of Great Britain and Northern Ireland (2 references); and countries in North America, such as Canada (4 references), Mexico (4 references), and the United States of America (6 references). An exception was Iceland (14 references). In total 39 countries made no references to the health impacts of climate change in their general debate address during this period.

Box 2 **Top 20 countries referencing climate change and health in speeches at the general debate of the United Nations General Assembly, 2000–2019**

Solomon Islands (28 references); Papua New Guinea (27 references); Palau (22 references); Federated States of Micronesia (22 references); Tuvalu (22 references); Trinidad and Tobago (21 references); Belize (18 references); Australia (16 references); Botswana (16 references); Saint Vincent and the Grenadines (15 references); Vanuatu (15 references); Iceland (14 references); Sierra Leone (14 references); Viet Nam (14 references); Mauritius (13 references); Eswatini (13 references); Ireland (12 references); Marshall Islands (12 references); Singapore (12 references); and Kiribati (11 references)

Notes: The references indicate the total number of separate mentions linking a key search term on health to a key search term on climate change in all of a country’s United Nations general debate statements between 2000 and 2019. The analysis considered 196 countries over the 20-year time period. The mean number of references to climate change and health for the full 20-year period was 4.52, with the number of references by countries ranging from 0 to 28. In total 39 countries made no references to the health impacts of climate change in their general debate address during this period.

**Fig. 2:**
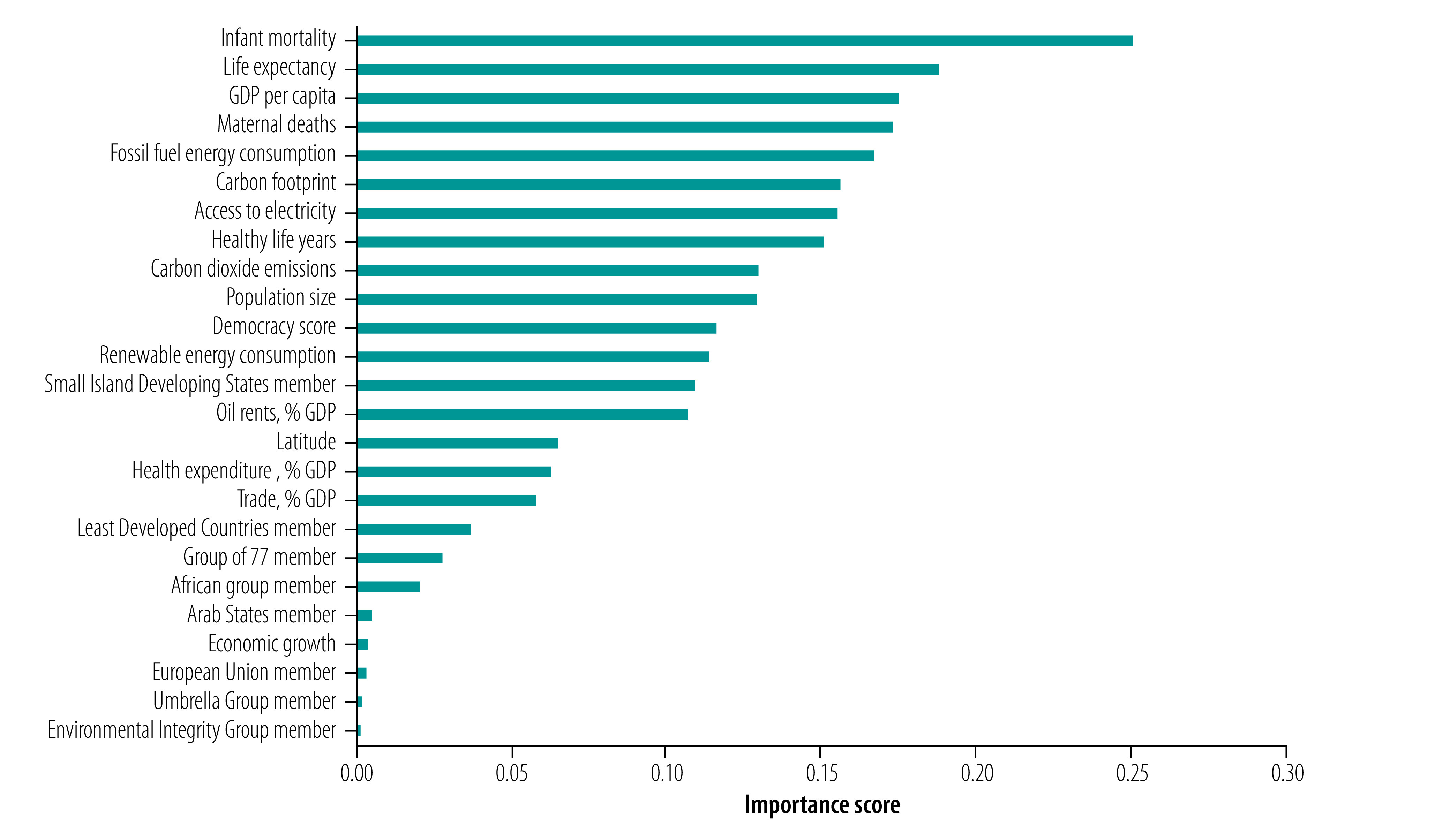
Importance of predictors of engagement with climate change and health in countries’ speeches at the general debate of the United Nations General Assembly, 2000–2019

Fig. 2 shows the results of the random forest prediction models, indicating the importance of the different predictors (more details provided in the data repository).^26^ We found that a country’s health profile was strongly associated with engagement with the health impacts of climate change in the UN general debate. Higher rates of infant mortality, maternal mortality and lower life expectancy were predictive of greater engagement. The permutation-based variable importance scores of the different predictors are: infant mortality score: 0.25; life expectancy score: 0.19; maternal mortality score: 0.17. Economic and emission-related factors (GDP per capita, fossil fuel energy consumption and carbon footprint) were also important predictors of engagement, with richer countries with higher energy consumption and larger carbon footprints being less likely to engage with the health impacts of climate change.

GDP: gross domestic product.

Notes: We used random forest permutations to determine importance scores. Number of trees: 5000; sample size: 3860 statements; number of independent variables: 25; variable importance mode: permutation; out-of-bag prediction error: 0.62; *R*^2^ (out-of-bag): 0.06. The figures show the permutation-based variable importance scores for each predictor, which captures average performance degradation of the model for each predictor, where the scale of the predictor importance is in terms of the out-of-bag prediction error. See Table 1 for definitions of predictors.

In contrast, we found that political groupings in the UN were much less important. The Small Island Developing States was the most important of the country groupings in predicting country engagement with the health outcomes of climate change (permutation-based variable importance score: 0.11), which is consistent with other studies that indicate that these states engage most with this issue in the UN.^15^ The results suggest that the Least Developed Countries, G77 and African Group of Negotiators groupings also had some influence. However, we found that membership of the Arab States grouping, the EU, the Umbrella Group and the Environmental Integrity Group were not predictors of the extent of countries’ engagement. 

### Nationally determined contributions

**Fig. 3:**
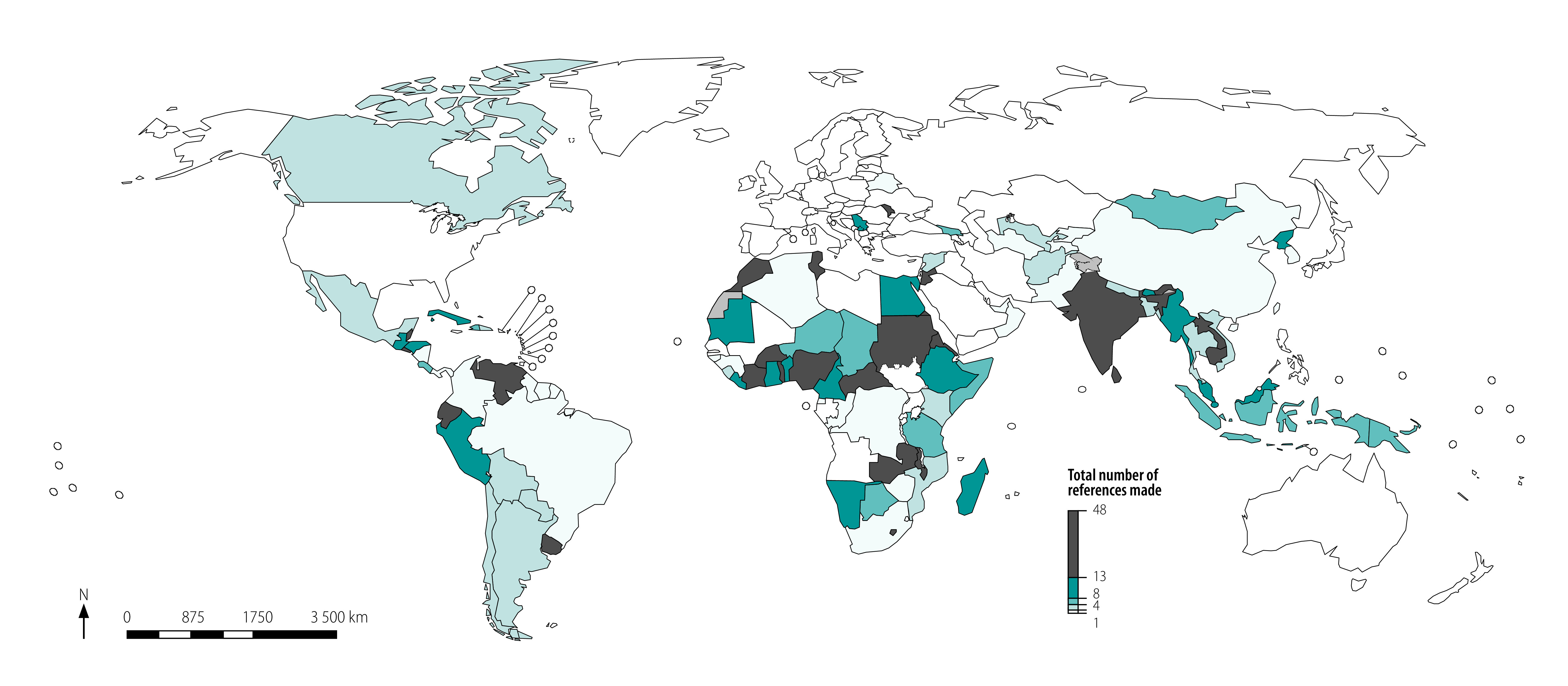
Countries’ references to health in nationally determined contributions agreed at the United Nations Framework Convention on Climate Change, 2016

We analysed nationally determined contributions of 185 countries. The majority of countries (135; 73%) referenced health in their pledges. However, in line with other studies, we found major differences in the extent of engagement based on our analysis of health terms, ranging from brief mentions of the general impact of climate change on health to detailed health adaptation plans.^15^ Fig. 3 (available at: http://www.who.int/bulletin/volumes/99/2/20-270033) shows there was a clear divide in engagement with climate change and health between the high-income countries and low- and middle-income countries. Low- and middle-income countries in Africa, Asia and Latin America had the highest levels of engagement with health in their nationally determined contributions. 

The same pattern can be seen in the list of 20 countries and territories with the highest number of references to health in their nationally determined contributions (Box 3). The mean number of references to health was 6.03, with the number of references by countries ranging from 0 to 46. Jordan has the highest number of health references (46 references), followed by Sri Lanka (40 references), Burkina Faso (34 references) and Ecuador (32 references). In contrast, we found no references to health in the nationally determined contributions of high-income countries such as Australia, the EU Member States, Japan, New Zealand and United States of America. In total, 50 countries made no reference to health in their nationally determined contributions.

Box 3 **Top 20 countries or territory referencing climate change and health in nationally determined contributions agreed at the UN Framework Convention on Climate Change, 2016**

Jordan (46 references); Sri Lanka (40 references); Burkina Faso (34 references); Ecuador (32 references); Republic of Moldova (32 references); Sudan (30 references); Seychelles (29 references); Lao People's Democratic Republic (27 references); Zambia (27 references); India (24 references); Belize (23 references); Uruguay (22 references); West Bank and Gaza Strip (22 references); Cambodia (21 references); Malawi (21 references); Nigeria (21 references); Eritrea (19 references); Morocco (19 references); El Salvador (18 references); and Timor-Leste (18 references)

Notes: The references indicate the number of separate mentions of the key search terms on health that are contained in the country’s nationally determined contribution. The analysis considered the nationally determined contributions of 185 countries. The mean number of references to health was 6.03, with number of references by countries ranging from 0 to 46. In total, 50 countries made no reference to health in their nationally determined contribution.

**Fig. 4:**
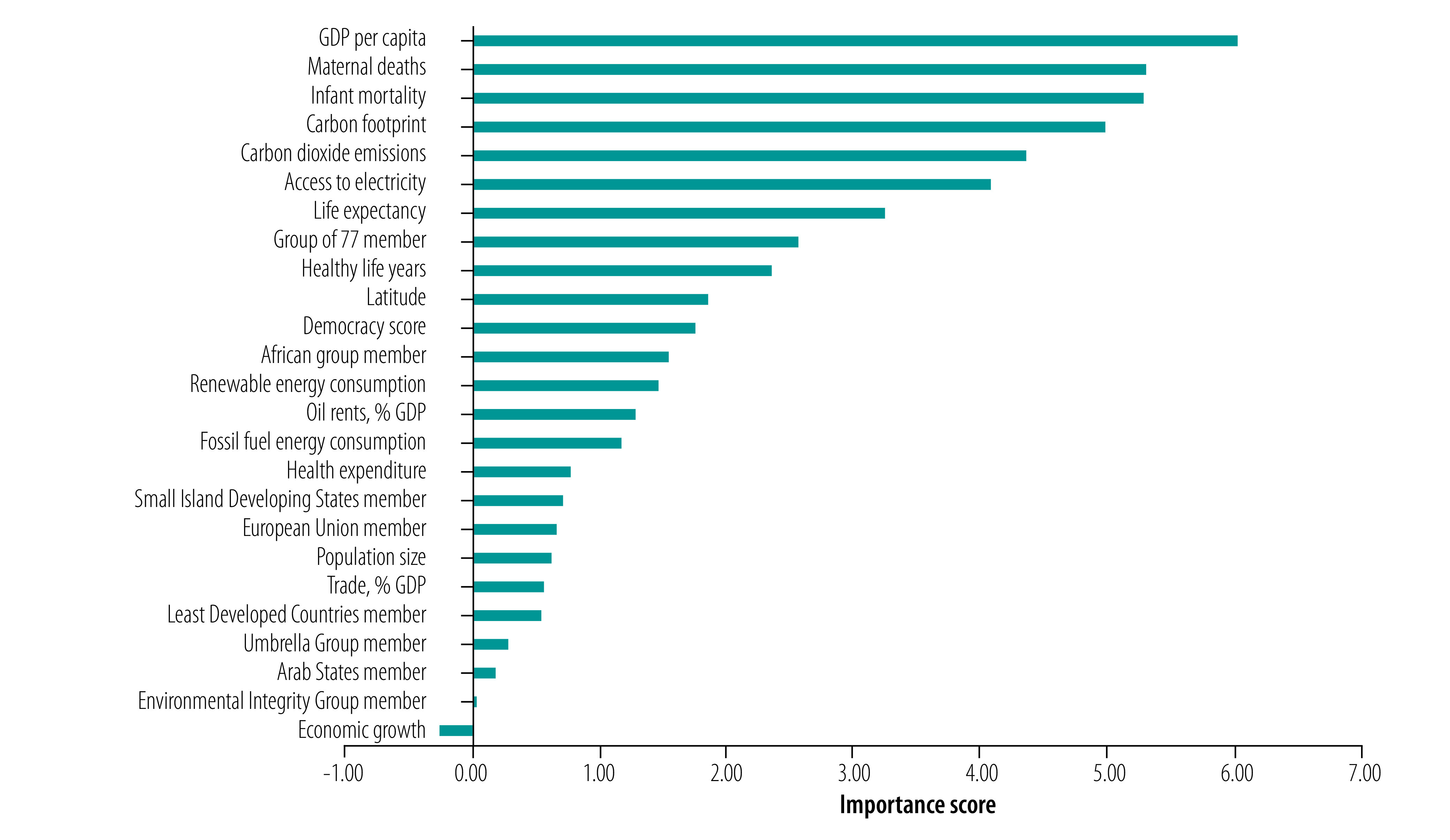
Importance of predictors of engagement with health in countries’ nationally determined contributions agreed at the United Nations Framework Convention on Climate Change, 2016

Fig. 4 shows the results of the random forest prediction models for engagement with health in the nationally determined contributions of 182 countries (data were missing for the predictors used in the model for three countries). There were similarities with the analysis of engagement in the UN general debate, with a country’s health, economic and emissions profile again predictive of engagement. GDP per capita was the most important predictor of whether countries engaged with health with a permutation-based variable importance of 6.02, followed by maternal and infant mortality statistics (which had permutation-based variable importance scores of 5.29 and 5.27). Countries’ carbon footprints, CO_2_ emissions, access to electricity and life expectancy were also important predictors.

GDP: gross domestic product.

Notes: We used random forest permutations to determine importance scores. Number of trees: 5000; sample size: 182 countries; number of independent variables: 25; variable importance mode: permutation; out-of-bag prediction error: 61.17; *R*^2^ (out-of-bag): 0.14. The figures show the permutation-based variable importance scores for each predictor, which captures average performance degradation of the model for each predictor, where the scale of the predictor importance is in terms of the out-of-bag prediction error. See Table 1 for definitions of predictors.

Among the country political groupings, the G77 and the Africa Group of Negotiators were more prominent as predictors of engagement with health in the nationally determined contributions. This finding suggests that membership of political groupings plays a more prominent role in explaining engagement with the health outcomes of climate change in countries’ mitigation and adaptation strategies than in their UN general debate statements. However, it is important to note that these groupings were far less important than factors such as GDP per capita, maternal death, infant mortality and CO_2_ emissions. The negative value for the economic growth variable suggests that a random permutation performed better in determining importance of the variable.

## Discussion

Our analysis showed differences in how much countries engage with the health impacts of climate change in their UN general debate statements and nationally determined contributions. We found evidence that low- and middle-income countries tended to give greater attention to the health impacts of climate change than did high-income and high-CO_2_-emitting countries. These differences were slightly less pronounced in the UN general debate statements than for the nationally determined contributions. This finding suggests greater variation in the health framing of climate change in countries’ climate change pledges – the central mechanism of global climate policy – than in other international fora such as the UN general debate.

Our study could only show associations, not causality, and was limited by the availability of data for the analysis of predictors. The study was based on only two structures within the UN; future research should examine engagement in health and climate change in a wider range of intergovernmental organizations. Nonetheless, both the UN General Assembly and the nationally determined contribution process are important arenas through which governments can make health central to global climate governance. Our analysis of these key settings for climate action provided little evidence that engagement with the health impact of climate change is occurring in either of the fora. This finding may change as countries prepare and submit their enhanced nationally determined contributions, which they are expected to do by the end of 2020.^35^ Engagement with health and climate change may increase particularly in higher-income countries with evidence showing that climate hazards are taking an increasing toll on population health.^36^^,^^37^ However, our analysis does not suggest that a health framing of climate change currently overcomes the geopolitical divisions that have shaped international efforts to respond to the threat posed by climate change.^38^

Instead, our analysis suggests that these divisions are influenced more by differences in countries’ health outcomes, income levels and fossil fuel consumption, and less by membership of political negotiating groups in the UN. With the UN general debate statements, we found that engagement with the health impacts of climate change were most predicted by countries’ infant mortality, life expectancy and GDP per capita. For the nationally determined contributions, the most important predictors were GDP per capita, risk of maternal death and infant mortality.

Put simply, it is those countries whose economies and lifestyles have contributed least to climate change that are shouldering responsibility for reminding the global community of the implications of climate change for people’s health. As many of these countries are among the most climate-vulnerable and poorest countries, they are carrying the heaviest health burden but lack the resources to address the accelerating impact of climate change.^1^^,^^2^^,^^6^ Our analysis suggests that it is this global inequality that is driving differences in the extent to which countries engage with the health impacts of climate change in international institutions. A potential risk is that the health consequences of climate change continue to be marginalized in the international political agenda and in the global governance structures needed to address these effects.

## References

[1] Watts N, Amann M, Arnell N, Ayeb-Karlsson S, Belesova K, Boykoff M, et al. The 2019 report of The Lancet Countdown on health and climate change: ensuring that the health of a child born today is not defined by a changing climate. Lancet. 2019 11 16;394(10211):1836–78. 10.1016/S0140-6736(19)32596-6 31733928 PMC7616843

[2] United in science: high-level synthesis report of latest climate science information convened by the Science Advisory Group of the UN Climate Action Summit. Geneva: World Meteorological Organization and United Nations Environment Programme; 2019. Available from: https://wedocs.unep.org/handle/20.500.11822/30023 [cited 2020 Jun 2].

[3] Lelieveld J, Evans JS, Fnais M, Giannadaki D, Pozzer A. The contribution of outdoor air pollution sources to premature mortality on a global scale. Nature. 2015 9 17;525(7569):367–71. 10.1038/nature15371 26381985

[4] WMO statement on the state of the global climate in 2018, WMO-No. 1233. Geneva: World Meteorological Organization; 2019. Available from: https://library.wmo.int/doc_num.php?explnum_id=5789 [cited 2020 Oct 1].

[5] WHO air pollution and child health: prescribing clean air. Geneva: World Health Organization; 2018. Available from: https://www.who.int/ceh/publications/air-pollution-child-health/en/ [cited 2020 Oct 1].

[6] Smith KR, Woodward A, Campbell-Lendrum D, Chadee DD, Honda Y, Liu Q, et al. Human health: impacts, adaptation and co-benefits. In: Field C, Barros V, Dokken D, Mach KJ, Mastrandrea MD, Bilir TE, et al., editors. Climate change 2014: impacts, adaptation, and vulnerability. Part A: global and sectoral aspects. Contribution of working group II to the fifth assessment report of the Intergovernmental Panel on Climate Change. Cambridge, UK: Cambridge University Press; 2014. pp. 709–54. Available from: https://www.ipcc.ch/site/assets/uploads/2018/02/WGIIAR5-Chap11_FINAL.pdf [cited 2020 Jun 2].

[7] Maibach EW, Nisbet M, Baldwin P, Akerlof K, Diao G. Reframing climate change as a public health issue: an exploratory study of public reactions. BMC Public Health. 2010 6 1;10(10):299. 10.1186/1471-2458-10-299 20515503 PMC2898822

[8] Costello A, Abbas M, Allen A, Ball S, Bell S, Bellamy R, et al. Managing the health effects of climate change. Lancet. 2009 5 16;373(9676):1693–733. 10.1016/S0140-6736(09)60935-1 19447250

[9] Watts N, Amann M, Ayeb-Karlsson S, Belesova K, Bouley T, Boykoff M, et al. The Lancet Countdown on health and climate change: from 25 years of inaction to a global transformation for public health. Lancet. 2018 2 10;391(10120):581–630. 10.1016/S0140-6736(17)32464-9 29096948

[10] Ziring L, Riggs RE, Plano JC. The United Nations: international organization and world politics. Boston: Cengage Learning; 2005.

[11] WHO review: health in the NDCs. Geneva: World Health Organization; 2019. Available from: https://www.who.int/publications-detail-redirect/who-review-health-in-the-ndcs [cited 2020 May 27].

[12] NDC explorer [internet]. Bonn: German Development Institute; 2018. Available from https://klimalog.die-gdi.de/ndc/ [cited 2020 May 10].

[13] Dickin S, Dzebo A. Missing in climate action: concrete health activities in nationally determined contributions. Lancet Planet Health. 2018 4;2(4):e144. 10.1016/S2542-5196(18)30046-9 29615212

[14] Workman A, Blashki G, Bowen KJ, Karoly DJ, Wiseman J. The political economy of health co-benefits: embedding health in the climate change agenda. Int J Environ Res Public Health. 2018 4 4;15(4):674. 10.3390/ijerph15040674 29617317 PMC5923716

[15] Watts N, Amann M, Arnell N, Ayeb-Karlsson S, Belesova K, Berry H, et al. The 2018 report of the Lancet Countdown on health and climate change: shaping the health of nations for centuries to come. Lancet. 2018 12 8;392(10163):2479–514. 10.1016/S0140-6736(18)32594-7 30503045 PMC7616804

[16] General Assembly of the United Nations. New York: United Nations; 2020. Available from: https://www.un.org/en/ga/ [cited 2020 Jun 14].

[17] Paris Agreement. New York: United Nations; 2015. Available from: https://unfccc.int/sites/default/files/english_paris_agreement.pdf [cited 2020 May 8].

[18] Baturo A, Dasandi N, Mikhaylov SJ. Understanding state preferences with text as data: introducing the UN General Debate Corpus. Research & Politics. 2017; (Apr-Jun):1–9. 10.1177/2053168017712821

[19] Smith C. Politics and process at the United Nations: the global dance. Boulder: Lynne Rienner; 2006.

[20] Tobin P, Schmidt NM, Tosun J, Burns C. Mapping states’ Paris climate pledges: analysing targets and groups at COP 21. Glob Environ Change. 2018;48:11–21. 10.1016/j.gloenvcha.2017.11.002

[21] Atteridge A, Verkuijlb C, Dzeboa A. Nationally determined contributions (NDCs) as instruments for promoting national development agendas? An analysis of small island developing states (SIDS). Clim Policy. 2020;20(4):485–98. 10.1080/14693062.2019.1605331

[22] Mills-Nova M, Liverman DM. Nationally determined contributions: material climate commitments and discursive positioning in the NDCs. Wiley Interdiscip Rev Clim Change. 2019;10:e589 10.1002/wcc.589

[23] Barnett J. The geopolitics of climate change. Geogr Compass. 2007;1(6):1361–75. 10.1111/j.1749-8198.2007.00066.x

[24] Kim SY, Russett B. The new politics of voting alignment in the United Nations General Assembly. Int Organ. 1996;50(4):629–52. 10.1017/S0020818300033531

[25] Party groupings [internet]. New York: United Nations Framework Convention on Climate Change; 2014. Available from: https://unfccc.int/process-and-meetings/parties-non-party-stakeholders/parties/party-groupings [cited 2020 May 8].

[26] Dasandi N, Graham H, Lampard P, Jankin Mikhaylov S. Supplementary webappendix: Country engagement with the health impacts of climate change in intergovernmental organizations [data repository]. Boston: Harvard Dataverse; 2020. 10.7910/DVN/QEFYO3 10.7910/DVN/QEFYO3

[27] NDC registry [internet]. New York: United Nations Framework Convention on Climate Change; 2020. Available from: https://www4.unfccc.int/sites/NDCStaging/Pages/All.aspx [cited 2020 May 29].

[28] Manning CD, Schütze H. Foundations of statistical language processing. Cambridge: MIT Press; 1999.

[29] World development indicators [internet]. Washington, DC: World Bank Group; 2020. Available from: https://data.worldbank.org/products/wdi [cited 2020 May 1].

[30] Hadenius A, Teorell J. Assessing alternative indices of democracy. C&M Working Paper. Lund: Lund University; 2005. Available from: https://portal.research.lu.se/portal/en/publications/assessing-alternative-indices-of-democracy(5a636298-5cf8-4c24-aa63-90e888b200d5)/export.html [cited 2020 May 27].

[31] Global burden of disease (GBD) [internet]. Seattle: Institute for Health Metrics and Evaluation; 2020. Available from http://www.healthdata.org/gbd [cited 2020 May 1].

[32] Data and methodology [internet]. Oakland: Global Footprint Network; 2020. Available from: https://www.footprintnetwork.org/resources/data/ [cited 2020 May 1].

[33] Breiman L. Random forests. Mach Learn. 2001;45(1):5–32. 10.1023/A:1010933404324

[34] Wright MN, Ziegler A. ranger: a fast implementation of random forests for high dimensional data in C++ and R. J Stat Softw. 2017;77(1):1–17. 10.18637/jss.v077.i01

[35] Fransen T, Northrop E, Mogelgaard K, Levin K. Enhancing NDCs by 2020: achieving the goals of the Paris Agreement. Working Paper. Washington, DC: World Resources Institute; 2017. Available from: http://www.wri.org/publication/NDC-enhancement-by-2020[cited 2020 October 1].

[36] Dodd W, Howard C, Rose C, Scott C, Scott P, Cunsolo A, et al. The summer of smoke: ecosocial and health impacts of a record wildfire season in the Northwest Territories, Canada. Lancet Glob Health. 2018;6 Special Issue:S30 10.1016/S2214-109X(18)30159-1 PMC6964492 29981098

[37] Carroll B, Morbey H, Balogh R, Araoz G. Flooded homes, broken bonds, the meaning of home, psychological processes and their impact on psychological health in a disaster. Health Place. 2009 6;15(2):540–7. 10.1016/j.healthplace.2008.08.009 18996730

[38] Barnett J. The geopolitics of climate change. Geogr Compass. 2007;1(6):1361–75. 10.1111/j.1749-8198.2007.00066.x

